# Crustal structure of Sicily from modelling of gravity and magnetic anomalies

**DOI:** 10.1038/s41598-020-72849-z

**Published:** 2020-09-29

**Authors:** M. Milano, Y. Kelemework, M. La Manna, M. Fedi, D. Montanari, M. Iorio

**Affiliations:** 1 Institute of Marine Sciences, National Research Council of Italy (CNR – ISMAR), Napoli, Italy; 2 University of Naples Federico II, DiSTAR, Napoli, Italy; 3 Institute of Geosciences and Earth Resources, National Research Council of Italy (CNR-IGG), Florence, Italy

**Keywords:** Geophysics, Tectonics

## Abstract

We aim at modeling the main crustal and thermal interfaces of Sicily (Italy), a key area for understanding the geological complexity at the collisional boundary between the African and European plates. To this end, we analyze the gravity and magnetic fields, integrated with information from well logs, geology, heat flow, and seismic data. In order to make the most accurate description of the crustal structure of the area, we modeled with different methodologies the carbonate and crystalline top surfaces, as well as the Moho and the Curie isotherm surface. The reconstruction of the carbonate platform is achieved using a nonlinear 3D method constrained by the available seismic and borehole data. The crystalline top, the Curie, and the Moho are instead estimated by spectral analysis of both gravity and magnetic data. The results show a complex carbonate basement and a deep crystalline crust in central Sicily, with a prominent uplift beneath the Hyblean Plateau. Maps of the Moho and the Curie isotherm surface define a variable thermal and structural setting of Sicily, with very thin crust in the southern and eastern sectors, where high heat flow is found, and deep and cold crust below the Caltanissetta Basin.

## Introduction

Sicily is a key-area for the interpretation of the Apennines-Tyrrhenian System. It is located in the center of the Mediterranean region, linking the Southern Apennines and the Calabrian Arc to the Tellian and Atlas systems of the Northeastern Africa plate^1–3^. This region is important for geothermal exploration, due to the widespread thermal manifestations at the surface, the medium-to-high heat flow and the thick Mesozoic carbonate basement, which may host an effective, low-to-medium temperature geothermal reservoir at a regional scale^4–8^. Accordingly, several geological, geochemical, seismic, GPS, heat flow surveys were performed in the attempt to understand the tectono-volcanic and dynamic evolution and for assessing the geothermal potential of the region (e.g. ^2,4,5,8^ and references therein). While the surface geology is fairly well known, the features of the deep crust are still debated^2,3,9–13^. A problem is that most previous studies inferring the deep crustal structure are related to sparse or spatial-limited data, such as seismic profiles and earthquake data sets. Potential fields are, instead, particularly suited to interpret complex geological scenarios, because of their full coverage of large regions.

In this study, we aim at providing a new comprehensive image of the main shallow and deep crustal boundaries of Sicily using potential field data: the carbonate basement is obtained by inversion of the vertical gravity gradient constrained with information from wells, geological models, and seismic profiles; the other crustal boundaries (crystalline basement, Moho boundary and Curie isotherm surface) are inferred from unconstrained spectral analysis of either gravity or magnetic field.

By carbonate basement top we mean the interface that separates Mesozoic carbonate rocks from synorogenic clastic deposits, allochthonous Sicilide nappes, and shallow sediments of alluvial or volcanic origin^14^. The crystalline basement broadly refers to the buried ‘subsurface’ of the covering sedimentary rocks. It is characterized by an increase in both density and magnetic susceptibility characterizing the transition from sedimentary to igneous/metamorphic rocks.

The Curie-Point Temperature (CPT) surface is defined as the magnetic-bottom crustal surface, depending on both the composition and the thermal features of the crust. Such a thermal boundary is usually associated with the magnetic properties of the magnetite, the most common magnetic mineral, which beyond ~ 580 °C has a ferromagnetic-to-paramagnetic transition (e.g.^15^). We point out that, as far as we know, a Curie depth model has not been yet published for Sicily.

The Moho, the boundary separating the Earth's crust from the mantle, is defined from seismic methods, which detect a rapid increase in seismic velocity at the crust-mantle interface. One of the main issues for seismic modelling the Moho is that seismic surveys have a limited areal extent. However, the Moho is also a density boundary. So, the gravity method represents a valid alternative to complete its modelling.

## Geological setting

Sicily belongs to the central-western part of the Mediterranean region, connecting the African Maghrebides with the southern Apennines, through the accretion wedge of Calabria (e.g.^10,12,13,16,17^). The geological and structural setting of Sicily is mostly associated with the convergence of the African and European lithospheres, with the counterclockwise rotation of the Sardinian-Corso block with the roll-back of the subduction margin of the Adriatic-Ionian-African plates and with the contemporary opening of the Tyrrhenian Sea (e.g.^16^).

**Figure 1:**
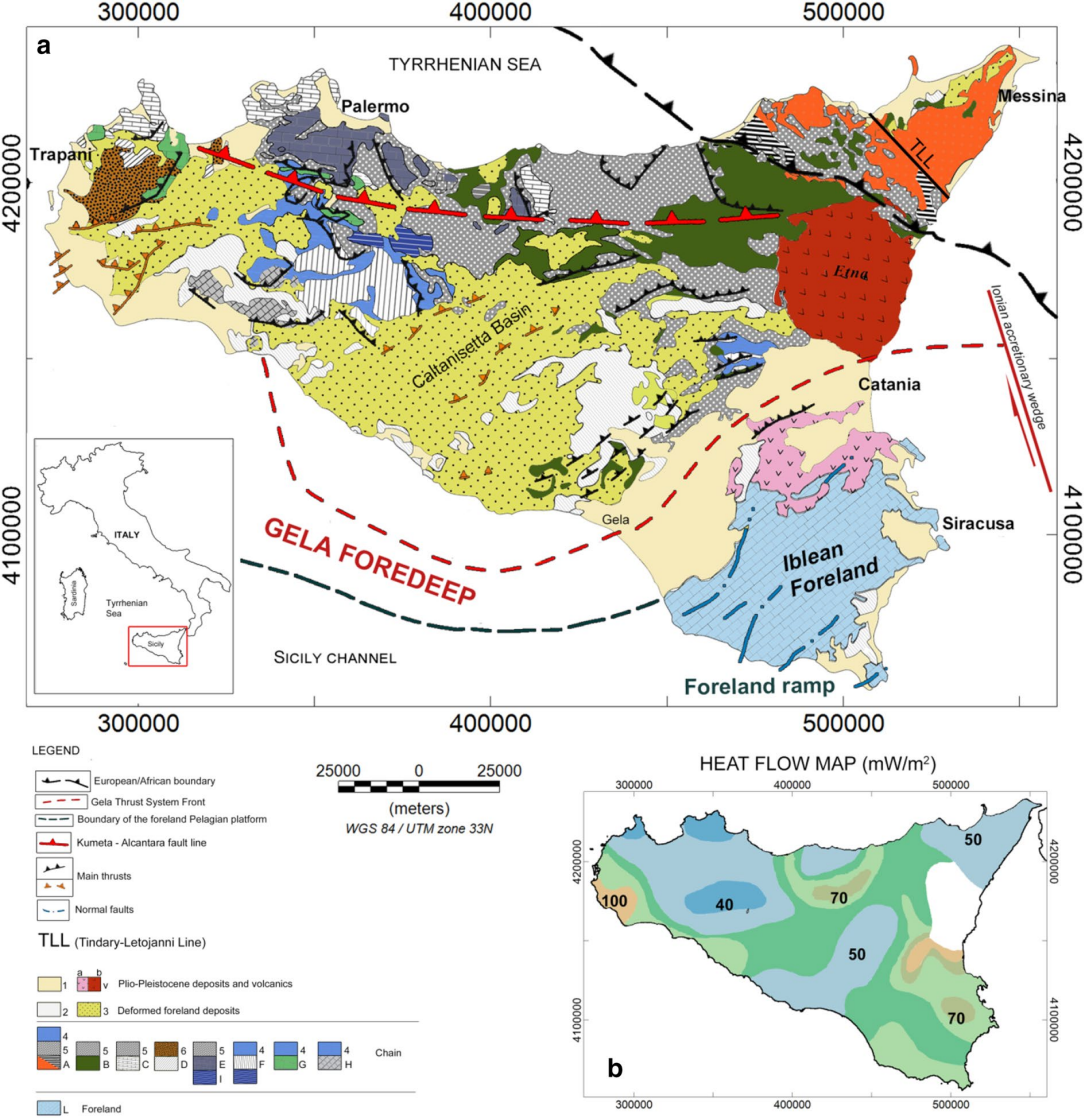
(**a**) Structural-geological map of Sicily [redrawn after^6,10,16,18,19^]; (**b**) Heat flow map of Sicily [redrawn from^4,7^]. 1, Pleistocene; 2, deformed foreland basin (Lower Pleistocene–Upper Pliocene); 3, Lower Pliocene–Upper Tortonian wedge top basin deposits (deformed foreland basin); 4, deformed foreland shelf margin (Middle to Lower Miocene); 5, Oligo-Miocene deformed foreland basin flysch units; 6, deformed foreland basin shelf margin (Lower Miocene–Upper Oligocene); A, Calabrian tectonic units (Oligocene–Paleozoic); B, Sicilide units (Oligocene–Upper Mesozoic); C, Panormide carbonate platform-derived tectonic units (Oligocene–Trias); D, pre-Panormide carbonate platform to basin-derived tectonic units (Oligocene–Trias); E, Imerese basin-derived tectonic units (Oligocene–Upper Mesozoic); F, Sicanian basin-derived tectonic units (Oligocene–Upper Mesozoic); G, Trapanese carbonate platform-derived units (Oligocene–Trias); H, Saccense carbonate platform-derived units (Oligocene–Trias); I, Lower Permian–Middle Triassic Lercara allochthons; L, Hyblean Pelagian platform units (Lower Pleistocene–Trias); V, volcanic rocks—(**a**) Pliocene, (**b**) Pleistocene. The maps have been produced using the software Oasis Montaj Geosoft (ver. 9.7.1; https://www.seequent.com/products-solutions/geosoft-oasis-montaj/).

The collisional complex of Sicily consists of three main regions (Fig. 1):

(i) a foreland area cropping out in the Hyblean Plateau and representing the onshore portion of the relative undeformed North Africa-Pelagian basement bounded to the east by the Ionian plate;
(ii) a tight Pliocene–Pleistocene NW-dipping foredeep, prolonging from SE to the Gela Basin;
(iii) the orogenic wedge made up of an E-SE trending fold and thrust complex^6,10,20–22^ made up of a European portion (Peloritani Units), a Tethyan element (Sicilide Units) and an African component (Maghrebian Sicilian Units)^2,3,9,20,23^.

The orogeny of Sicily mainly includes four geological units stacked above the original Hyblean-Pelagian platform^1–3^. The main bulk of the chain consists of a thick Meso-Cenozoic carbonate platform, with the overlying Imerese and Sicanian units, representing a wedge of deformed Meso-Cenozoic deep-water carbonate thrust sheets and over-thrusting the carbonate platform and the Hyblean foreland. Above the carbonate-level lays, the wedge of Sicilian nappes stacked over warped Oligo-Miocene Numidian flysch units (e.g.^24^). The fourth and higher level contains the Calabrian ‘backstop’ units consisting of uppermost Miocene–lower Pleistocene clastics.

In central Sicily, the most important structure is the Caltanissetta basin, a structural depression originated in the middle-late Miocene and filled by Pliocene–Pleistocene marine deposits and nappes, which progressively over-thrust each other and finally slide into the basin^25^. The Northern Chain extends along the Tyrrhenian boundary and consists of a stacking of south-verging thrusts. The Kumeta-Alcantara fault zone separates the Northern Chain from the Caltanissetta basin, while the Etna volcano is situated south of the intersection of the Kumeta-Alcantara and Tindary-Letojanni lines. The Hyblean Plateau is a dome-shaped promontory resulting from the flexural mechanisms of the Pelagian platform during the southeastward migration of the Sicilian orogenic wedge (e.g.^26,27^). This region is controlled by a system of major extensional faults on its margins, with considerable vertical offset (e.g.^28^). It is mainly made up of thick carbonate successions of deep-water Mesozoic-Cenozoic rocks^24^, overlaid by Late Cretaceous‐Late Miocene outcrop sedimentary units^26^. Magmatic volcanism also deeply affected the Hyblean area, as a result of crustal extension^29,30^. Several eruptive centers associated with late Cretaceous, late Miocene and Plio-Pleistocene cycles (e.g.^26^) gave rise to extended volcanic products intercalated with the carbonate sedimentary units, as revealed by the deep borehole surveys. Moreover, vertical motions and progressive uplift of the Hyblean Plateau have been directly associated with upper crust magmatic and diapiric intrusion kinematics^31,32^.

## Geophysical background

While the surface geology is fairly well known, the features of the deep crust are still debated (e.g.^2,3,9–13,33^). Many geophysical studies have been conducted to interpret the deep subsurface structure and the volcano-tectonic evolution of the region, focusing on the interpretation of *seismic data* (e.g.,^34–38^), *gravity data* (e.g.^6,32,37,39^) and *magnetic data* (e.g.^1,40^).

In particular, deep seismic investigations led to construct geological cross-sections extending from western to eastern Sicily (e.g.^1–3,6,16,18,22,24,41,42^). Deep seismic soundings (DSS) and wide-angle reflection/refraction profiling (WARRP) sections^34,36,43–45^ were performed since the 70s, providing information on the main crustal units and discontinuities beneath the northern border of the continental Sicily. The SI.RI.PRO project^37^ aimed at investigating the crust of the Sicilian orogen from the Tyrrhenian margin to the Sicily Channel with a multidisciplinary approach involving seismic reflection and gravity methods^6^. The resulting geological cross-section confirmed the crustal structures already outlined in previous studies.

As regarding the deep structures, geophysical studies (e.g.^6,37,40,46^), based on seismic and potential field data, provided several information on the Crystalline Basement Top (CBT) and the Moho depth beneath Sicily. These studies however suffer from insufficient seismic coverage of the region, or for low detail. Further studies of the deep crustal structures have been achieved again from seismological data analysis in Sicily (e.g.^45,47,48^). These authors carried out crustal velocity models by using depth distribution and kinematic info from hundreds of seismological focal mechanisms. These models show the main interfaces of the crust and identify the thickness of the main crustal units.

Preliminary interpretation based on the SI.RI.PRO seismic transect across central Sicily has associated the crystalline basement top to a reflector at 7 s TWT, corresponding to a 14–16 km depth below the surface^37^. Later, a new interpretation pointed out the crystalline layer having variable thickness^6^, with a progressive deepening from South (~ 10 km) to North (~ 20 km). In particular, the authors recognized a considerable depression of the CBT in correspondence with the Caltanissetta basin (~ 25 km). However, further studies proposed a slightly different crystalline top surface after performing a re-processing and signal-to-noise ratio improvement of the seismic reflection stack^46^. Their CBT depth is relatively shallow at the south-easternmost area (~ 12/14 km) and increases rapidly below the Caltanissetta depression (~ 24 km), while rises again in the northern sector (~ 19.5 km). More recently, the seismic data have been re-processed using a wave equation datuming technique and provided a new image of the CBT along the SI.RI.PRO transect, reaching the maximum depth beneath the Caltanissetta basin, at around 21–22 km ^38^.

In addition, seismological studies (e.g.^48^) have shown that the shallowest portion of the crystalline crust consists of a metamorphic Permo-Trias basement, with the top around 12 km depth underlying the stack of sedimentary and Mesozoic carbonate rocks. Such interpretation demands to a crystalline basement considerably shallower than that found from seismic data analysis.

The Moho boundary in Sicily has been investigated in detail over the last decades, by integrating information from seismic surveys, gravity and seismological data and structural geology (e.g.,^3,6,37,38,41,43,46,48^). Wide-angle reflection/refraction seismic data were carried out from surveys in 1971^49^, 1973^50^ and 1985^51^, indicating a Moho around 20 km deep in the offshore regions, deepening down to 25 km depth at the South-Western coast^41^ and reaching 40 km depth in the central part of the Caltanissetta trough. Moreover, it was suggested a direct correlation between the Moho deepening toward central Sicily and the strong gravity low anomaly^43^. Also, in northern Sicily the Moho was estimated deep, at about 37–38 km^34,49^, progressively rising to 10 km depth in southern Tyrrhenian. In the south-eastern region, beneath the Hyblean platform, seismic data were interpreted with a very thick crust down to 35–40 km depth, showing a rapid uplift of the Moho along the Ionian margin, around 16 km depth, and deepening down to 20 km depth at a greater distance from the coast (e.g.^35,41,52,53^). Additional results have been achieved by the processing of the SI.RI.PRO data. Earliest studies (e.g.^6,37^) identified the Moho at about 14 s TWT in northern Sicily, say ~ 38 km, with a progressively rise toward the Hyblean foreland at 12 s and 9 s TWT, say around 25 km. The results of the data re-processing show a Moho located at ~ 32 km below the Hyblean foreland, relatively flat in the central part (~ 35 km), and rapidly increasing in depth to the North (> 40 km)^46^. Finally, the Moho was interpreted as slightly dipping from the foreland (~ 30 km) to the Tyrrhenian coast (~ 38 km)^38^. Deep values of the Moho in central Sicily have also been achieved by seismological data analysis^48^ where 1-D velocities models suggest this boundary at around 37 km depth. Using gravity modeling constrained by seismic and petrophysical data, the Moho depth was found varying from 16 to 17 km beneath the Tyrrhenian Coastline of Sicily, 30 km beneath the Peloritani Mountains, and about 20 km beneath the Etna volcano^54^.

Heat flow^4^ shows a variable distribution, which reflects the geological complexity of the region (Fig. 1b). To the North, the heat flow reaches high values around 120 mW/m^2^ above the Tyrrhenian area and considerably decreases toward central Sicily (40–60 mW/m^2^), where a thick crust has been observed from seismic data. High heat flow values have been measured at depth in oil and gas wells at the southwestern coast (60–100 mW/m^2^)^55^. Moreover, further authors mention the occurrence of moderately high heat flow areas in the same region^4,8^. On the other hand, higher values associated with the recent magmatic and volcanic activity are observed in the eastern regions of Sicily, as well as in the thin Tyrrhenian oceanic crust.

## Data and methods

Previous studies of potential fields were carried out^40^ to build 2D forward models from gravity and magnetic data, integrating the available information from structural geology. These studies suggested a complex map of the crystalline basement ranging from about 4 to 10 km SE to about 8–10 km in the western sector of Sicily and deepening down to 17 km beneath the Caltanissetta trough^1,40^.

Our crustal modeling of the Sicily regards the 3D interpretation of the whole maps of gravity^56^ and magnetic^57^ data. We obtained the gravity map of Sicily by merging the offshore gravity measurements^50^ with the onshore dataset^56^, so obtaining a 222 × 430 grid with a 2 km step-size. In detail^58^, the complete Bouguer gravity dataset was obtained adopting the following parameters: (i) a constant density of 2.67 g/cm^3^ for the Bouguer slab reduction; (ii) the international formula 1980 (IAG80) for the theoretical gravity; (iii) a 2nd order free air reduction; (iv) a terrain correction extended to a radius of 166.736 km at each measurement station using a digital elevation model.

**Figure 2:**
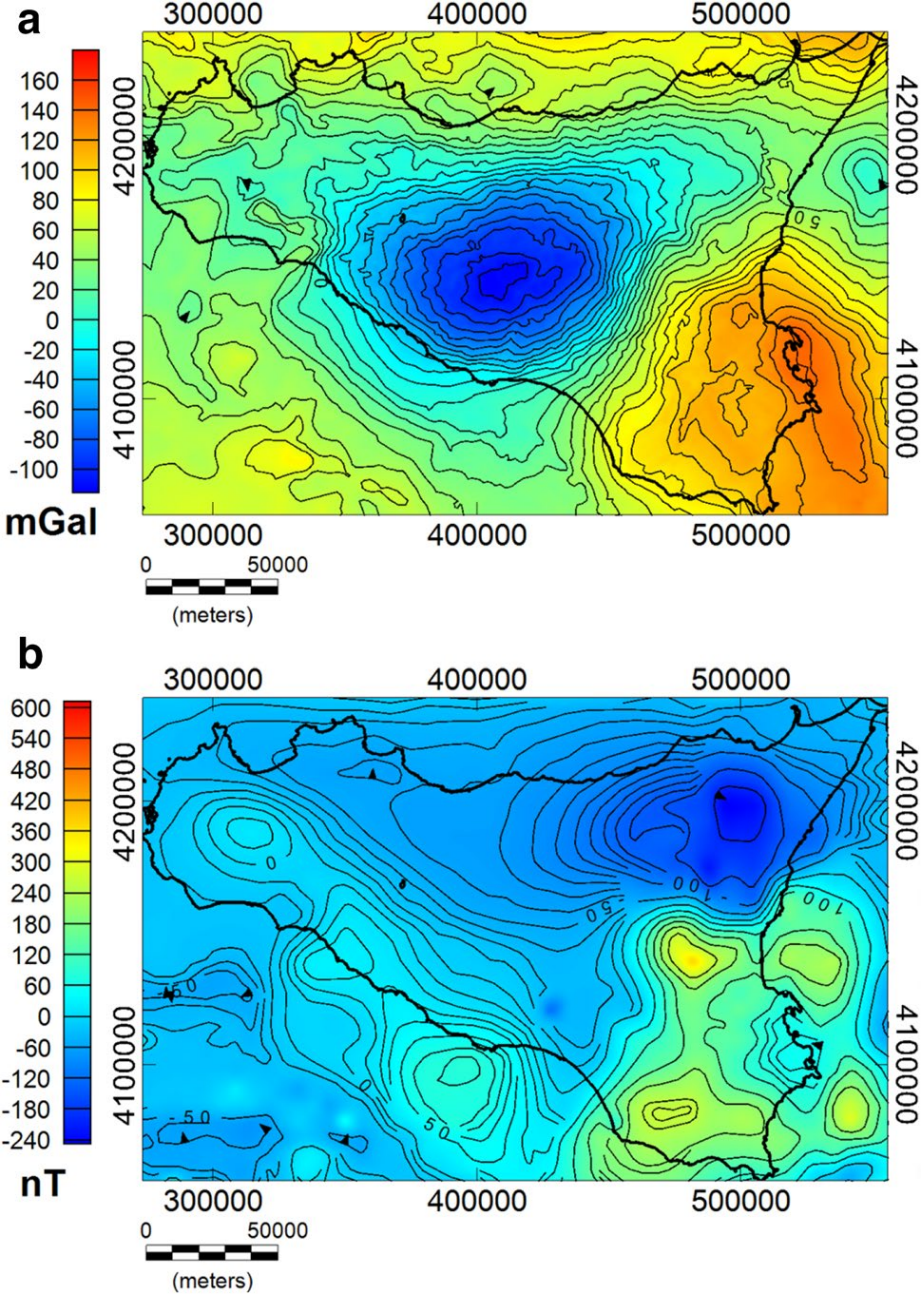
Bouguer gravity field (**a**) and aeromagnetic field map (**b**) above Sicily.

As regards the magnetic field, we used aeromagnetic data of Italy acquired between 1971 and 1980 by AGIP^57^. The dataset was compiled by a total of 265,305 km of survey lines with a 2 km step size. The magnetic anomaly field map was obtained after the subtraction of the regional field (AGIP Reference Geomagnetic Field)^59^. Unfortunately, the data relative to the Etna region are not reliable because of the measurement altitude (2130 m), lower than most of Mount Etna's relief. Therefore, the field analyzed here is the residual obtained after subtraction of the Etna anomaly, which has been removed locally with a special technique based on the discrete wavelet transform^60^. The maps of the gravity and magnetic data used in this study are shown, respectively in Fig. 2a and b.

A qualitative comparison between the two maps shows a substantially different behavior of the anomaly fields. This difference can be attributed to the fact that Sicily is a region affected by diffuse volcanism and magmatic intrusive bodies which strongly contribute to the magnetic anomaly field (e.g.^32^). In fact, the magnetic field is highly sensitive to volcanic and igneous rocks, whose susceptibility contrast with surrounding carbonates is very high. Conversely, the corresponding density contrast is not relevant.

This can be observed, for instance, in the offshore region of the Ionian Sea, along the south-western coast of Sicily, and above the Hyblean foreland, where areas of intrusive and volcanic activity are known (e.g.^26,29,30^). On the other hand, the Bouguer gravity field map (Fig. 2a) demands to a strong correlation with large-scale and deep structures and less to local density contrast. The gravity field is indeed mostly characterized by long-wavelength anomalies and the main contribution is certainly a strong and extended low related to the Caltanissetta Basin, in central Sicily, which can be probably associated to a prominent depression involving the lower portion of the crust, as a consequence of the northerly dipping Hyblean-Pelagian platform and the SE-verging thrust system (e.g.^18^).

As we already noted, we choose two specific methodologies to analyze potential fields: the 3D nonlinear interface inversion and the spectral analysis.

Modeling of the Mesozoic carbonate basement was achieved by using a nonlinear inverse approach^61^, which consists of evaluating a surface representing the top of a homogenous layer (e.g., the carbonate basement) formed by a set of adjacent homogenous prismatic sources (see “Methods” for detail). The method does not require any a priori knowledge of density (magnetization) contrast, while information about the maximum and minimum depth of the basement morphology is needed. Obviously, the method is well suited to explore the carbonate basement, because the above two constraints may often be reasonably assumed, for instance by wells information and seismic data.

At larger depths, we used spectral methods. We computed the spectra from the total magnetic field at 2130 m altitude and from the vertical derivative of the gravity field. This last was upward continued to 3.2 km above the mean sea level and low-pass filtered for wavelength less than 14 km to remove residual high-wavenumber noise due to vertical differentiation. Due to the large altitudes of the datasets and to the low-pass filtering, the analyzed fields present not reliable high-wavenumber content. So, spectral methods will yield unconstrained depth estimates at large depths. In particular, we estimated: (a) the morphology of the crystalline basement top from both gravity and magnetic data; (b) the bottom of the magnetic crust, i.e., the Curie isotherm, from magnetic data; (c) the Moho boundary from Bouguer gravity anomalies. Different spectral techniques may be used for depth estimation, assuming either a statistical ensemble of blocks^62–64^, a random source distribution (e.g.^65–69^), or even a fractal source distribution (e.g.^70–75^). Spectral methods provide valid results if the statistical source model is adequate for the studied region and optimal window size is chosen for the range of presumed depths^63,76^. Though there is still no agreement on the minimum extent required to get a reliable depth to the bottom estimate, window size should be large enough to capture deep anomalies and small in high heat flow and volcanic regions (e.g.^66,77^). Depending on crustal structure and geological complexity of a region, a window size (3 to 5 times the expected depth) may provide reliable results, as already adopted in many studies (e.g.^75,77–81^) for the centroid and modified centroid methods. The nonlinear inversion method requires a relatively large window size (i.e. 10 times the expected depth or more)^73^, while the de-fractal method a window size greater than 5 times the expected depth^82^. Different window sizes over different geological provinces have been adopted^83^ to improve the performance of the spectral peak method.

Specifically, we used the statistical block-ensemble model^62,63^ (see “Methods”). We estimated the depth to the source by radial spectra computed within a running window. We choose windows with variable size, mainly because the geologic setting of Sicily varies significantly. For example, the southeastern part of the Tyrrhenian Sea is characterized by relatively elevated heat flow (> 100 mW/m^2^) (e.g.^4^) and a Moho depth as shallow as 20 km. Therefore, by using a large window, i.e. greater than 80 km, we would incorporate anomalies from completely different neighboring regions (i.e. Sicily and Calabria), which could affect the estimate of the Curie depth in both regions. On the other hand, the central part of Sicily is associated with low heat flow (40–60 mW/m^2^) and the Moho depth is lying at depths greater than 30 km (e.g.^35,38^), which seems to indicate a large depth for the Curie isotherm in this region. Thus, a window size of about 90 km or more may provide reliable results with good resolution. Finally, the Sicily channel rift zone is characterized by relatively high heat flow (60–100 mW/m^2^) (e.g.^4^) and a Moho depth from 20 to 25 km (e.g.^84,85^), which suggest a window size of about 80 km or more. For this heterogeneity, we avoided a uniform window-overlapping, differently from what is commonly adopted in most of the published works.

With all the previous cautions in mind and to partially improve the problem of mixing different geologic provinces, we considered different window sizes in a reasonable way. Specifically, we applied an 80 km × 80 km with a 30 km overlap over the southern Tyrrhenian Sea and Sicily channel rift zone, Hyblean plateau and western Sicily, and 90 km × 90 km with 35 km overlap over the central Sicily. Examples of power spectra used to estimate the depth to the top and bottom are shown in Fig. S1 (see Supplementary Material).

## Results

### Carbonate basement modeling

Mesozoic carbonate rocks are the main target for the exploration and exploitation of low-to-medium enthalpy geothermal systems in Italy (e.g.^7,8,86–89^).

**Figure 3:**
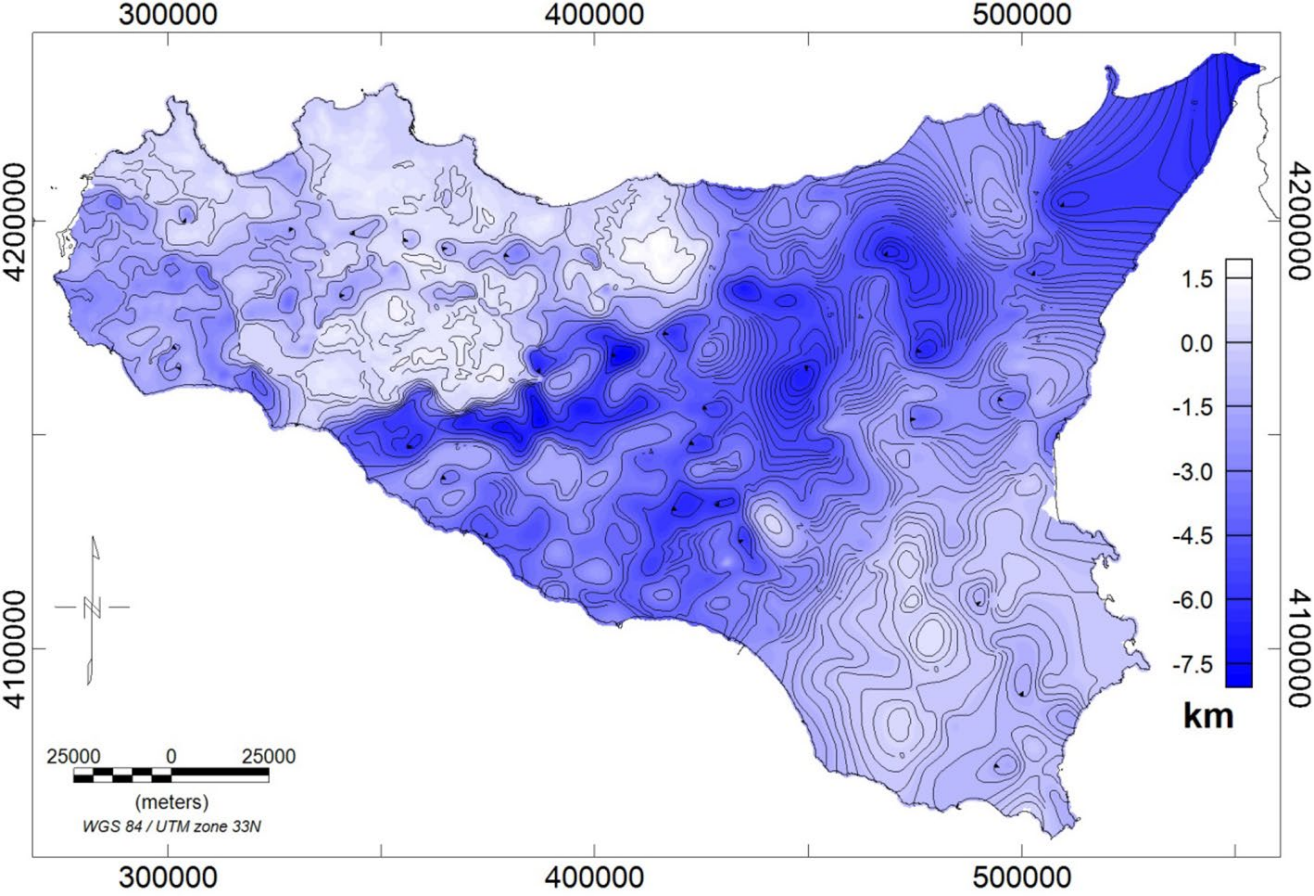
Carbonate top surface of Sicily (meters b.s.l.) obtained from gravity, seismic, and well logs data (modified after^7^).

A map of the carbonate top (Fig. 3) was published by^7^, by integrating well-data^90,91^, seismic sections (e.g.^92–94^; ViDEPI project website https://www.videpi.com), and gravity-based depths of the basement. Gravity data are indeed particularly useful for a detailed reconstruction of the carbonate top surface and yield a continuous description that seismic data and wells information cannot necessarily provide. We illustrate here the results of the inversion of the gravity data with the nonlinear method proposed by^61^.

**Figure 4:**
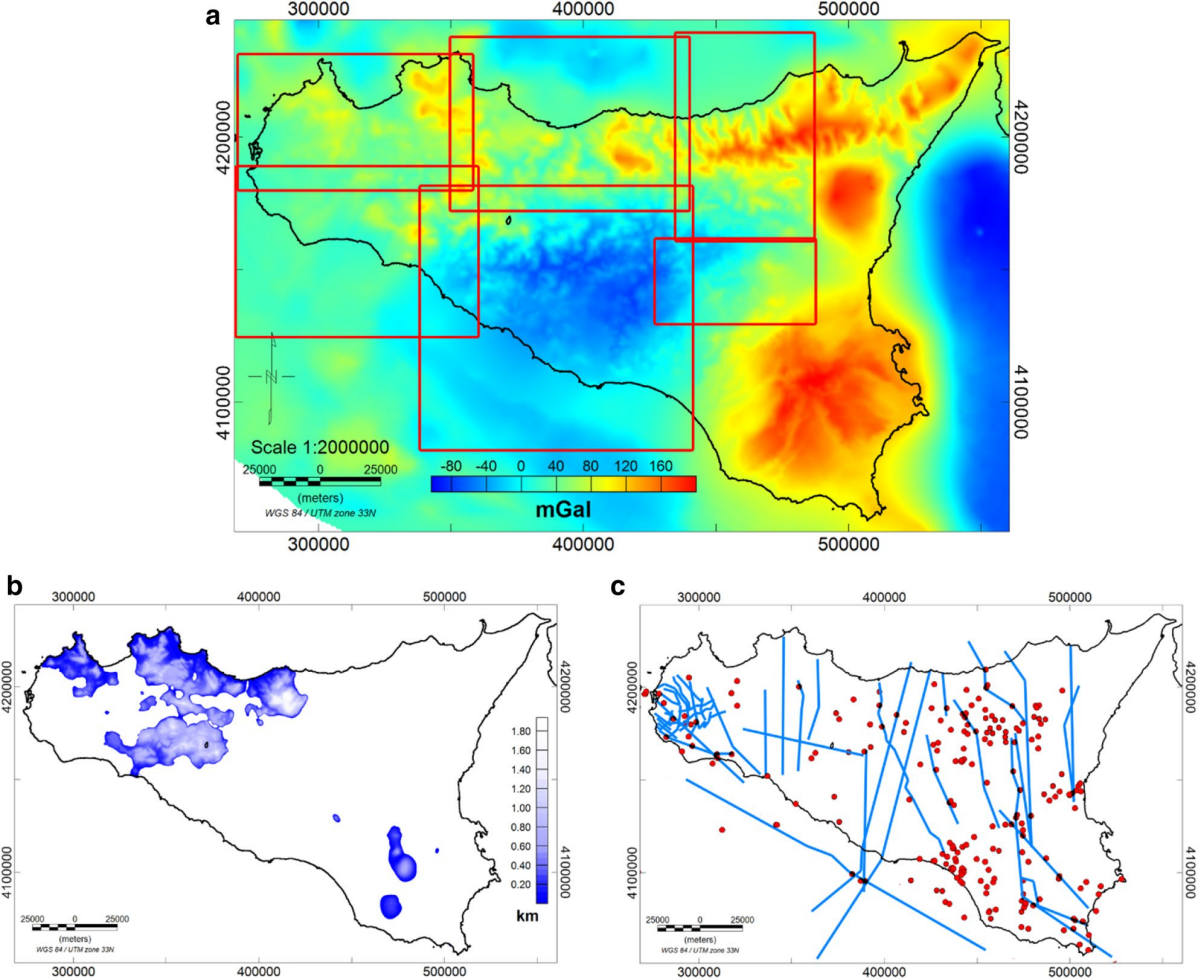
(**a**) Free-air gravity field map with the subareas selected to compute the carbonate basement (red rectangles); (**b**) Geological data of the outcropping Mesozoic carbonates; (**c**) well logs (red dots) and seismic profiles (blue lines) used to constrain the carbonate basement model.

First of all, being the carbonate basement partially outcropping, one may wonder whether using free-air or Bouguer gravity data. The choice depends strongly on the geological setting of the area. Indeed, it is well known that Bouguer slab reduction removes most of the gravity effect related to the topography. Therefore, where the carbonate basement is deep and buried beneath other rocks (i.e. marls and marly-clays, volcanic or others), as in the Caltanissetta Basin in central Sicily, it is necessary to work with a Bouguer anomaly dataset, in which the effect of the reliefs, other than carbonates, is minimized. On the other hand, in regions where the carbonate is very shallow or outcropping, the effect of the topography must not be removed, since they are part of the surface to be estimated, and free air gravimetric anomalies are preferred. Moreover, the measured field is the sum of effects from different depths and the density contrast may vary, even horizontally: so, a single interface could not be the most appropriate hypothesis. Therefore, the region has been divided into subareas, in each of which a “single interface” hypothesis can be considered reasonable (Fig. 4a). The vertical gradient of the gravity field has been used to reduce the effects from the deep sources and to increase those from the shallow ones.

Thus, the inversion of the gravity vertical gradient was performed for each subarea in two phases: the first was to identify the outcropping carbonate deposits (Fig. 4b), characterized by a very high-density contrast, while the second was carried out to identify the buried basement characterized by lower density contrast. Our nonlinear inversion needs two depth-constraints for each subarea: we fixed the shallowest depth from well logs or outcrop information, and the deepest one from well logs or by seismic profiles (Fig. 4b,c).

**Figure 5:**
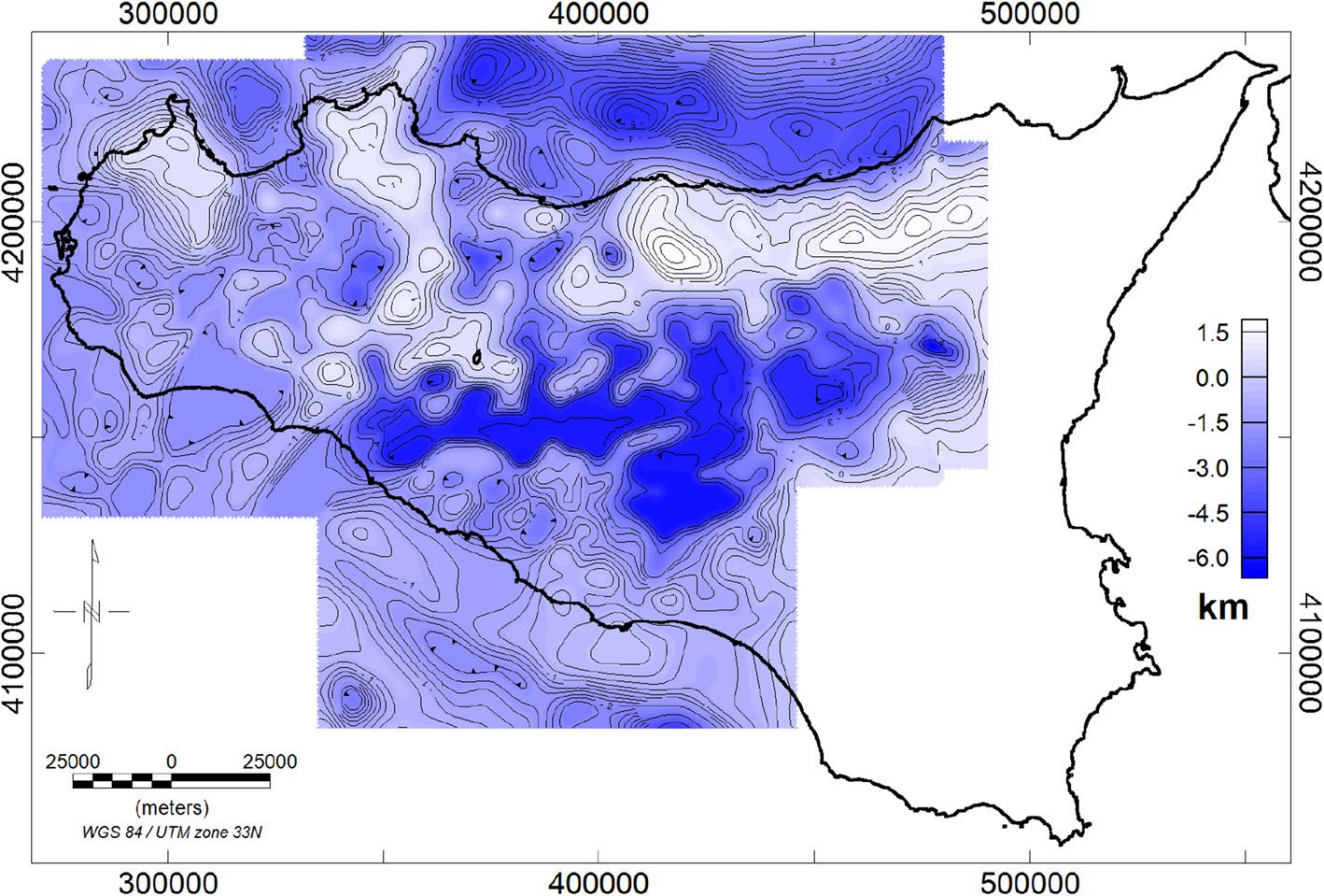
Carbonate top surface obtained from free-air and Bouguer gravity data inversion.

The estimated carbonate basement for all the subareas was then merged by using the GridKnit function of the Oasis Montaj Geosoft software, to produce a single surface covering most of the Sicilian region (Fig. 5). Some areas in eastern Sicily have not been included, since the complex geological setting does not allow achieving reliable models of the carbonate basement: these areas, indeed, are made up of an intricate stack of carbonate and volcanic rocks with density value mostly similar, so it is not possible to define a single surface for a specific lithology. This task is even more difficult in the region of the Hyblean Plateau, where Mesozoic carbonate units are underlying quaternary carbonate rocks, as confirmed by the deep borehole investigations. Moreover, upper crust magmatic intrusions are producing strong density contrast with the embedding carbonate rocks (e.g.^32^).

**Figure 6:**
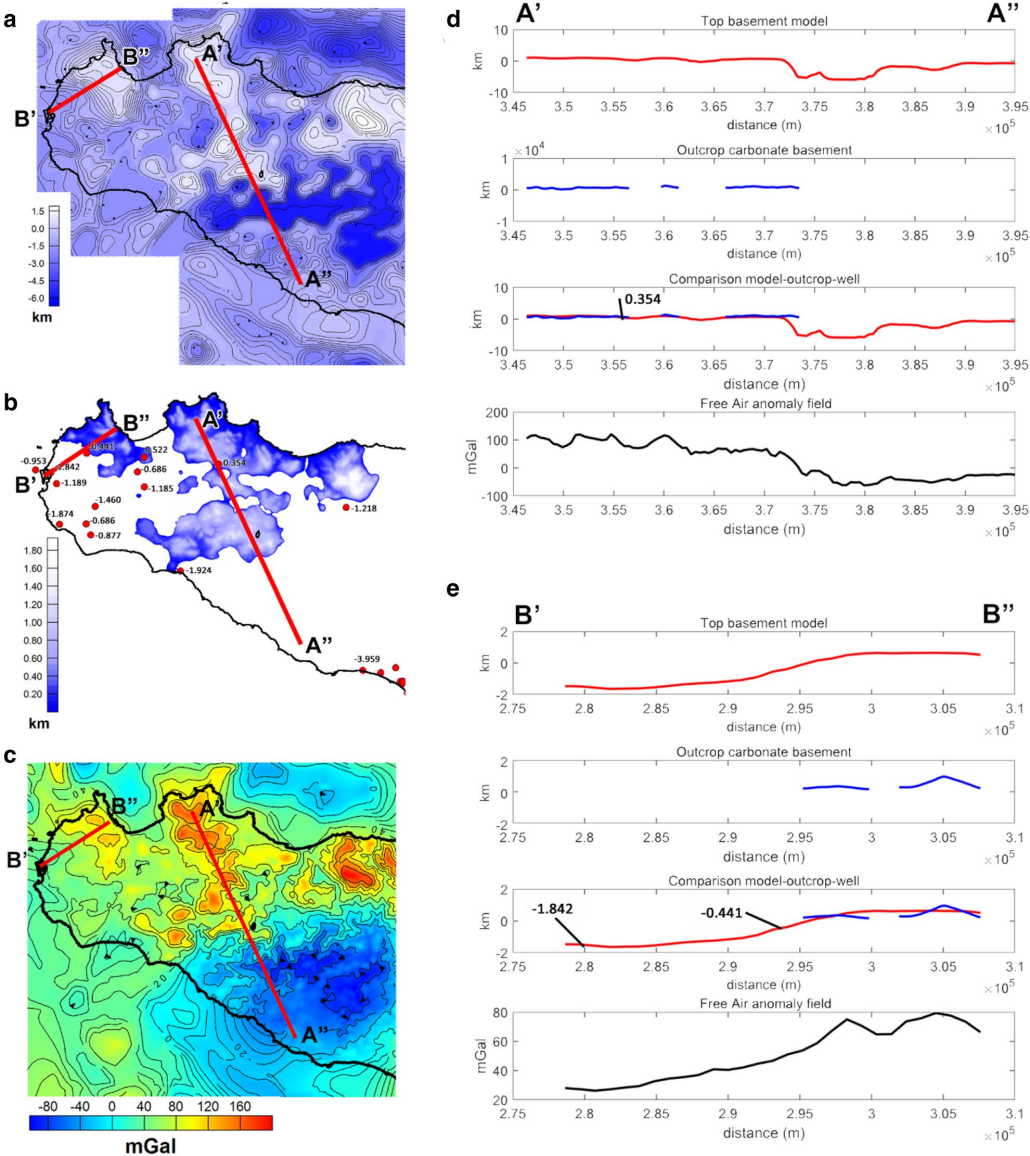
Localization of profiles A’A” and B’B” on the carbonate top surface map represented with carbonate basement depths relative to the well logs (**a**), the outcrop Mesozoic carbonate map (**b**), and the Free Air gravity map (**c**); Comparison between the model profile A’A” (**d**) and B’B” (**e**) with wells, outcrop data, and Free-air anomaly data along the same profiles.

As mentioned above, we compared the estimated results with the depths of the carbonate identified by the well logs. A quantitative comparison has been possible where well-data reaches the top of the Mesozoic basement (Fig. 6a). In Fig. 6c and d, we compare the profiles A’A” and B’B”, extracted from the model map, with the carbonate outcrop data (Fig. 6b) and the depth of the well (Fig. 6a) located along with the same profile. In other regions, where the well-drilling did not intercept the carbonate top, we found that the modeled surface is consistently deeper than the maximum drilling depth. A general good agreement between the model and outcrop/wells depths can be observed, obviously within the limits of the resolution of the available gravity data.

### Crystalline basement modeling from gravity and magnetic data

Different models have been proposed for defining the geological setting of the Sicilian Island and surrounding regions, based on structural and stratigraphic data collected throughout Sicily over many years integrated with geophysical and borehole data. Even though the overall stratigraphy and shallow crustal structure are fairly known, there is still a high degree of uncertainty regarding the depth and extent of the bottom of the carbonate basement/top of the crystalline basement of the region.

**Figure 7:**
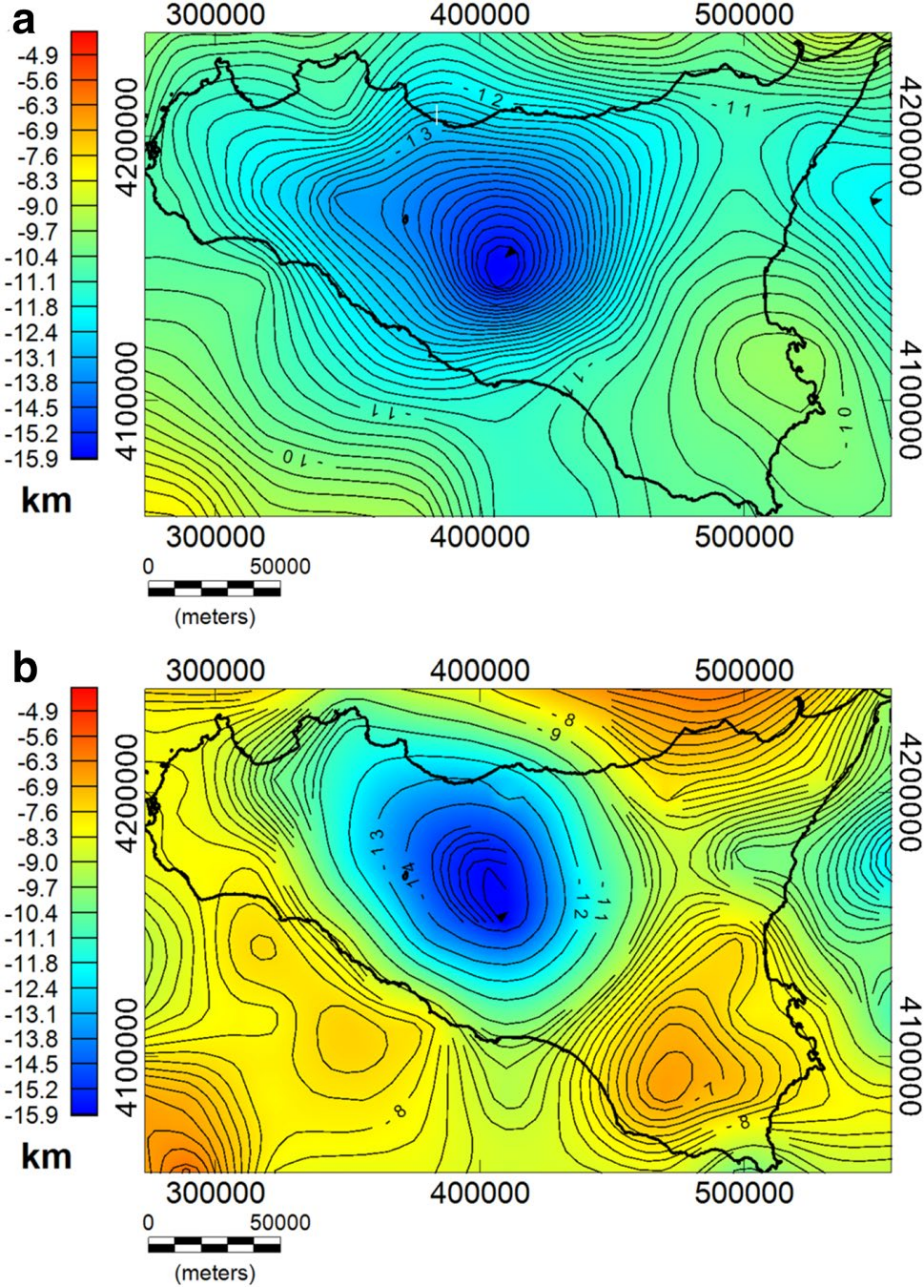
Maps of the crystalline top obtained from the spectral analysis of gravity (**a**) and magnetic (**b**) data.

Here, we present the maps of the crystalline basement of whole Sicily derived from the spectral analysis of both gravity (Fig. 7a) and magnetic (Fig. 7b) data. The depth estimates are obtained after correcting for the reference altitude of the datasets; hence, the estimated depths are relative to the mean sea level. These models show a variable morphology characterized by a shallow depth beneath the southwestern offshore of Sicily and a large depth in the central part, toward the Caltanissetta basin. In both maps, we found a similar trend in the mainland of Sicily but also some discordance on the offshore and external regions. A large basement depth (~ 16 km) is found beneath the Caltanissetta Basin, progressively decreasing to about 10–13 km towards the north coast. This prominent depression is found both in gravity and magnetic models and is in accordance with previous interpretations (e.g.^1,40^). The depth to the top of magnetic sources beneath the Sicily channel is ranging from 5 to 10 km with a NW–SE trend. This rift zone is characterized by the occurrence of widespread Late Miocene to Quaternary volcanic activity, continuing up to historical times (e.g.^95^). Moreover, this region is known to be long affected by significant tectonic stretching, which is manifested by several depressions (i.e. the Pantelleria, Malta, and the Linosa grabens) bounded by NE-SW trending normal faults (e.g.^85,96^, and references therein), as well as by diffused magmatic activity. The depth to the top of the basement estimated from gravity data ranges from 8 to 12 km, which is deeper than the range estimated from magnetic data, but in good agreement with the previous 2D deep crustal model of gravity data along with several CROP seismic profiles and across the Sicily channel^85^.

Besides the general agreement between the two maps in the average trend of the crystalline top, some discrepancies are also observed in areas where there are volcanic rocks and possible intra-sedimentary magmatic intrusions in the upper crust. As previously mentioned, these structures can produce a consistent contrast of magnetization with the surrounding rocks which could possibly affect the estimation of the magnetic basement top. This is particularly visible in the Sicily channel zone and Hyblean plateau, where, we see in both maps an uplift of the crystalline top, but with different depths values. Moreover, the different locations of the maximum uplift between the two maps can be reasonably associated with the presence of strongly magnetized intra-sedimentary bodies within the Hyblean upper crust. This is also found by other authors (e.g.^32,38,97,98^) who pointed out that the deep Hyblean crust may be constituted of different layers of re-crystallized magmatic rocks and that magnetic intrusion spreading and thermo-metamorphic processes may have occurred in the region. Such an explanation can be reasonably extended over other regions, such as in the Sicily Channel and Gela foredeep region, where several magmatic manifestations have occurred (e.g.^99^).

On the other hand, at a regional scale, the local effect of intrusive bodies can be negligible for the gravity field, due to the generally low-density contrast between carbonate and igneous rocks. Therefore, we are more confident with the crystalline basement obtained by the gravity field analysis, which is less affected by local-scale effects, compared to the magnetic surface. Nevertheless, we remark that both contain interesting information for understanding the crust properties.

### Depth estimation for Moho and Curie isothermal surface

**Figure 8:**
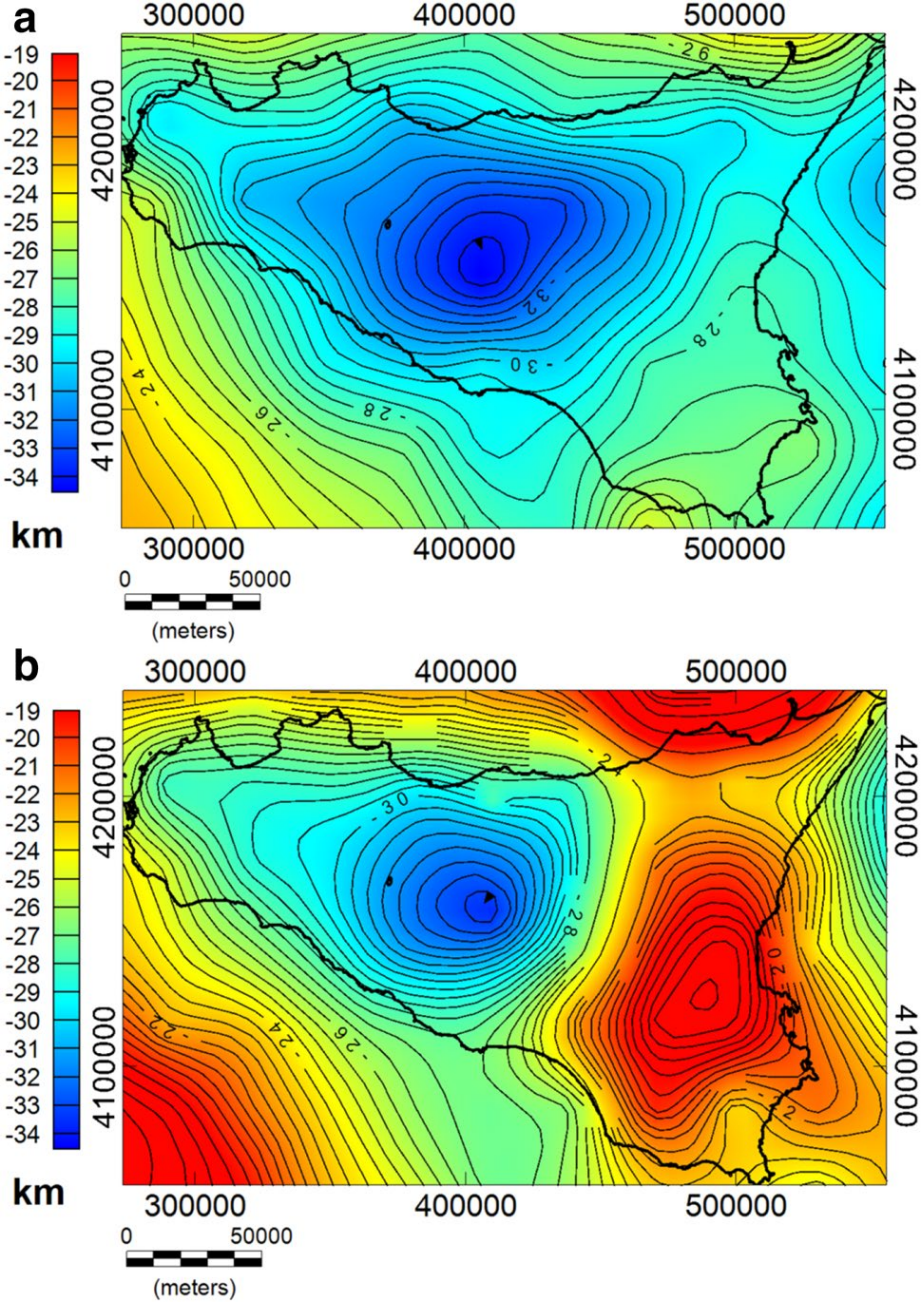
Maps of the gravity-based Moho boundary (**a**) and the magnetic-based Curie Isotherm (**b**) beneath Sicily.

We show the estimated depths to the Moho from gravity data and the Curie isothermal surface from magnetic data in Fig. 8a and b, respectively. The Moho ranges from 22 km depth beneath the Sicily channel and the Sicily-Southern Tyrrhenian boundary to 35 km depth beneath the Caltanissetta Basin, while the Curie isothermal surface ranges from 10 to 34 km depth.

Although the Curie isothermal surface described in Fig. 8b have a smoother variability than heat flow measurements, the Curie isothermal surface and heat flow (e.g.^4^; Fig. 1b) show correlations that suggest that higher heat flow values are located in sectors with shallower Curie depths. Specifically, a NW–SE trending region of shallow Moho depth, 22 km is observed beneath the Sicily channel (i.e. the Pantelleria graben, Linosa graben, and Malta graben), which progressively decreases to about 28 km towards the coast (Fig. 8a). This region is characterized by a shallow depth to the bottom of magnetic sources, ranging from 14 km depth in the Pantelleria, Linosa, and Malta graben to about 26 km depth at the coast of Sicily. In this area, we have a relatively high heat flow, up to 100 mW/m^2^ (e.g.^4^), which well correlates with the computed shallow Curie temperature isotherm. Other seismic and gravimetric studies of crustal structure also revealed a shallow thickness of about 20 km beneath the Sicily channel, gradually thickening toward the Sicilian coast (e.g.^38,84,85^).

Shallow depths (~ 12 km) are observed beneath the southeastern part of the Tyrrhenian Sea, which indicates a rising of Curie temperature isotherm (Fig. 8b). This shallow Curie temperature isotherm corresponds to high heat flow values, up to more than 100 mW/m^2^^4^. The Moho depth is found to be slightly deeper (20 km) (e.g.^38,100^). Comparing to other geological and geophysical data, our estimated Curie depth points describe the expected model, where the shape of the isothermal surface is strictly related to the crustal heat flow.

The gravity-based Moho boundary beneath the Hyblean Plateau is estimated at 27–30 km depth. This region is recognized as a relatively undeformed foreland of the collisional zone, which is acting as resisting block indenting the Maghrebian–Apenninic chain (e.g.^38^). The Moho uplift observed in the map is in accordance with^32^, where the gravity positive anomaly of southeastern Sicily was interpreted as possibly associated with a Moho uprising as a consequence of a Mesozoic rifting. The Curie isotherm beneath this foreland is also found to be shallow, 16–20 km, and it is consistent with the relatively high heat flow value (e.g.^4^).

## Discussion

The obtained results are here discussed and compared with seismic sections along the mainland Sicily and offshore (Fig. S2a in Supplementary information). As said above, we aim at providing a regional-scale interpretation of these structural boundaries, thanks to the good areal coverage given by potential field data. However, some differences could occur among them because: (a) spectral analysis depth estimations have a resolution limited to the window size, while seismic reflectors may be locally have a higher resolution; (b) gravity anomalies depend on density, while seismic reflection data on seismic velocities, thus the two kinds of data are relative to different physical parameters.

As regards the carbonate top basement, we find that our model well agrees with seismic/geological interpretations, as well as with borehole data (Fig. 6d,e). This is observed, for instance, in central Sicily, along with the SI.RI.PRO seismic transect (Fig. S2b). In particular, we find a good correspondence of our top surface with the Meso-Cenozoic Imere units shown in the SI.RI.PRO geological model^6^.

Regarding the crystalline top, we compared the surfaces obtained by gravity and magnetic data with an existing model from mainly magnetic data^40^ and with seismic sections. An overall correspondence is observed with our results, i.e. at the Caltanissetta basin and the Hyblean foreland, but we find also some differences. The previous crystalline basement map shows a prominent uplift in western Sicily and the southern shoreland^40^. We find similar features in our model inferred by magnetic data (Fig. 7b), but the crystalline model obtained by gravity is instead rather different (Fig. 7a), being considerably smoother and deeper. We explain this difference as due to shallow igneous rocks and intra-sedimentary magmatic intrusions which contribute strongly to the magnetic anomaly field (e.g.^101^) and, therefore, affect the estimation of the crystalline basement top. Thus, we believe that the gravity model would be more reliable, being gravity data less affected by local such intra-sedimentary features.

A comparison with seismic data shows a general agreement in the southern offshore Sicily. Our results have been indeed compared with the CROP M-25^102^, the Ministerial seismic lines G82-150, G82-103, and G82-153 and with the gravity models^99^ carried out along the same seismic profiles. The authors, indeed, show a crystalline basement with a density of 2.83 g/cm^3^ at depths ranging between 10 and 12 km b.s.l., which is in good accordance with our model obtained by gravity data (Fig. S2b).

In western Sicily, our crystalline top model is found deeper than that interpreted by seismic data (e.g.^1,2,16,42^). Our model, indeed, shows the crystalline surface progressively deepening in the central area (~ 12 km), as being a westward prolongation of the Caltanissetta depression. The seismic section, instead, shows a shallow crystalline surface at around 6–7 km depth^16,42^. However, the seismic profiles are mostly focused on the carbonate units and its complex thrust system, while the deep crystalline surface appears less well imaged.

In central Sicily, our results have been compared with the geological interpretation of the SI.RI.PRO seismic transect^6,38,46^ (Fig. S2b) and with interpretation carried out by seismological data^48^. Our model shows a gently dipping basement surface below the Caltanissetta basin, reaching depths around 15 km, that is in rather good agreement with the results of the seismological data. The SI.RI.PRO models, instead, shows a deeper crystalline crust, also affected by an orogenic wedge which is overlying the autochthonous Hyblean units (e.g.^6^). The complexity of the whole area will probably deserve further discussion and integrated analysis.

As regards the computed Moho boundary (Fig. 8a), we firstly compared our results with the Moho depth^38^ drawn from seismic reflection interpretation^35^ and earthquakes tomography analysis^103–105^. We found our model almost similar, consisting in three main domains: (i) a relatively shallow Moho in the eastern region; ii) a deep Moho in the central region reaching depths > 35 km; (iii) a progressively crustal thinning in the offshore area to the south-west. Both the gravity-based Moho depth and the maximum depth of the magnetic sources (Curie isotherm surface) are estimated at more than 35 km deep beneath the central Sicily and the depth gradually decreases to about 30 km below the coastline and in western Sicily, which denotes a lower thermal gradient of the crust beneath the Caltanissetta Basin.

The SI.RI.PRO seismic transect in central Sicily revealed a complex Moho architecture consisting of a progressive dipping below the foredeep^106^ and a reverse fault offsetting of the Moho as consequence of the southern Tyrrhenian mantle wedge accretion (e.g.^6^). By comparing this seismic model^6^ with our Moho estimates along the same profile (Fig. S2b in the Supplementary material), we observe an overall agreement in the southern-central part, where the depth to the Moho varies between 30 and 35 km. Our results show a smooth Moho boundary that reaches its maximum depth below the Caltanissetta basin. Our model is not well consistent northward, where an accretionary mantle wedge has been proposed^6^. It rather supports a continuous Moho interface not affected by a reverse fault offsetting, in agreement with other studies^107^, which also discarded a vertical Moho offsetting and the assumption of the mantle wedge.

Beneath the Sicily channel rift zone, depth estimates of the Moho and CPT surface are found to be shallow, with the Moho lying at less than 22 km and the CPT as shallow as 15 km (Fig. S2c), in agreement with previous studies^85^. CROP seismic data confirmed that this zone is dominated by a NW–SE-trending system of right-shear faults and associated NNE-SSW left-strike-slip faults^3^. The shallow spectral depth estimates together with the positive Bouguer anomalies and relatively elevated heat flow, further confirm the hypothesis that the crust is thinning in this region.

There is a clear correlation between the crystalline top and the Moho boundary surfaces. To this regard we observe that both the crystalline basements from gravity and magnetic fields present independently similar trends (Fig. 7a,b). On the other hand, the Moho and the Curie isotherm surface also show independently such crustal depression. We remember that the Curie isotherm surface in a low heat-flow region (the Caltanissetta basin) is expected to resemble the Moho depth. Thus, taking into account that these results come from independent analyses of two very different geophysical quantities we are confident in being the Moho and crystalline basement rather correlated.

Finally, we give some information about the uncertainty of the spectral depth estimates used to build the crystalline, Moho and CPT surfaces. To this end we used Eqs. (13) and (14) (see “Methods”) and found depth errors varying from 0.6 to 1.8 km for the crystalline top and ranging 2.3–4 km for the Curie isothermal surface and Moho boundary.

## Conclusions

Potential fields are important to interpret shallow and deep crustal structures of a region, thanks to the unique information deriving from their complete coverage of measurements. This is particularly true where direct information or other geophysical information is lacking. By these methods, we have here studied the Sicily Island, which represents an exceptional laboratory, characterized by a complex geological and geothermal setting.

We show that gravity data are particularly useful to successfully reconstruct the surface of the carbonate top. The resulting model was constrained by the available geological data of the outcropping carbonate, well-log and seismic data. Unfortunately, many well logs have not been utilized because not intercepting the carbonate surface; nevertheless, our model is consistent and indicates in these areas a deeper carbonate basement top. As expected, the main limitation is that gravity modeling is unable to identify the boundary between geological units having similar density values, as observed in the Hyblean region.

Regarding the deepest structures, the spectral analysis on both gravity and magnetic fields confirmed to be a powerful technique of depth estimation, which is independent on a-priori information from other data. We have shown that the crystalline top as well as the Moho and the Curie-isotherm surfaces may be successfully estimated using variable window sizes for different geological provinces. We also pointed out that gravity field data are more suitable to model the crystalline basement top in regions affected by volcanism and intrusive magmatism. The maps obtained from gravity and magnetic data show indeed some differences which have been addressed to the intense contribution of highly magnetized intra-sedimentary bodies within the upper crust, which may affect sensibly the estimation of the magnetic basement surface. The crystalline depth map obtained by gravity data is, instead, smoother and less sensitive to the effects of intra-sedimentary intrusive bodies.

Modeling of the Moho and Curie isotherms has been a difficult task, because of the complex geological scenario and the presence of extended volcanic and hot crustal provinces. The choice of appropriate windows size is crucial since, in regions of high heat flow and low crustal thickness, a large window extent could include anomalies from the neighboring regions and consequently affect the estimation of the Curie and Moho depth in both areas. On the other hand, a relatively large window size was adopted above cold areas, where sources are expected to be particularly deep. Our gravity-based Moho depth provides a new image of the gravity Moho over the whole Sicilian territory and a valid upgrade of previous models where seismic surveys are not available. The Curie-isotherm surface model of Sicily shows a variable thermal setting of Sicily, in good accordance with the estimated heat flow offshore and onshore.

This study shows that both gravity and magnetic methods may be considered as efficient techniques for characterizing structural and lithologic changes resulting from significant subsurface density and magnetization contrasts. Moreover, we believe that our modelled deep structures, such as the Curie isotherm surface, could represent a valid contribution to the understanding of the geothermal potential of Sicily, which is among the most geothermically promising regions in Italy.

## Methods

### Inversion for the carbonate top surface

**Figure 9:**
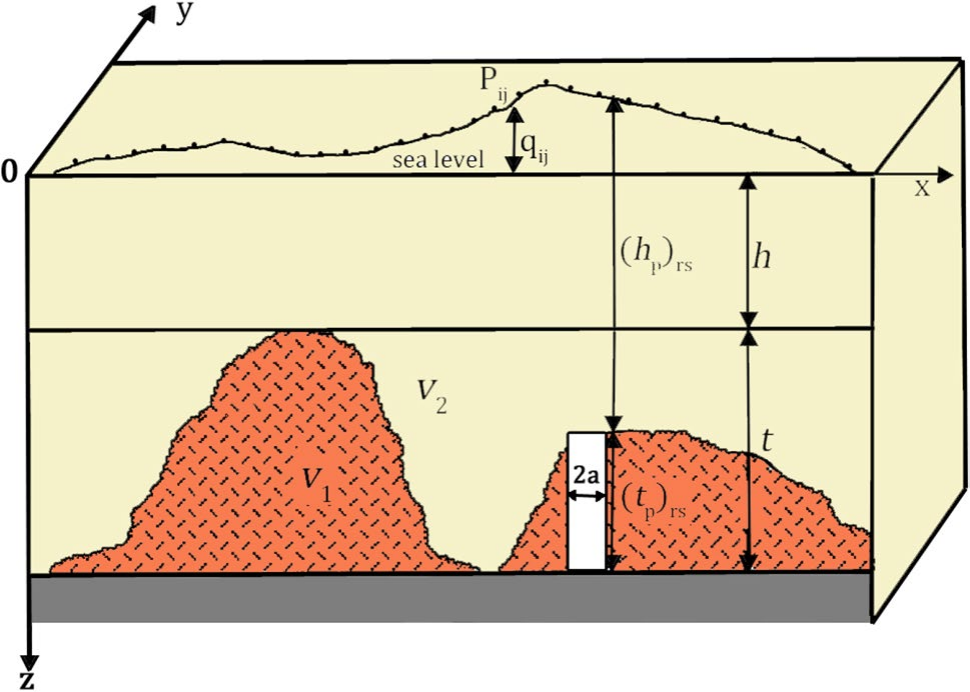
Sketch model of an interface separating two media producing a density contrast *v* = *v*_1_ − *v*_2_. The model is composed of a set of prisms with a lateral extent of 2a and thickness ${\left({h}_{p}\right)}_{rs}$(modified after^61^).

Here we briefly describe the methodology used to compute the carbonate top model from gravity data. For a complete review and a detailed description of the theoretical formulation, we refer to the original paper^61^. According to the author, an interface separating two media of different magnetization or density can be computed assuming a homogeneous body composed of a set of adjacent prisms with variable depth to the top and thickness (Fig. 9). Thus, the gravity field due to the effect of each prism (${G}_{ij}^{rs}$) can be expressed as:

1 $$G_{ij} = \mathop \sum \limits_{r = 1}^{M} \mathop \sum \limits_{s = 1}^{N} G_{ij}^{rs} \left[ {v, \left( {h_{p} } \right)_{rs} , \left( {t_{p} } \right)_{rs} , a, b, h, t} \right]\;\;\;\;\;\;i, r = \left\{ {1, \ldots , M} \right\}; j, s = \left\{ {1, \ldots , N} \right\}$$

where ${G}_{ij}$ is the gravity field measurement of a discrete set of *M* × *N* data at points (${P}_{ij}$), with a step size of 2*a* and 2*b* along the *x* and *y* axes; *v* is the density (or magnetization) contrast between the prisms (*v*_1_) and the surrounding rock (*v*_*2*_); ${h}_{p}$ and ${t}_{p}$ are, respectively, the depth to the top and the thickness of a prism with constant horizontal size 2*a* × 2*b*. The inverse problem consists of determining ${h}_{p}$ and *v* from the data ${G}_{ij}$ assuming that ${\left({h}_{p}\right)}_{rs}$ can vary between *h* and *h* + *t*, which are fixed as constraints. Therefore, a-priori information is needed to constrain the shallowest depth to the top and the maximum thickness of the layer where the interface to find is contained.

By using such a-priori constraints, Eq. 1 can be expressed in terms of apparent densities (${\Psi }_{ij}$) of an equivalent layer of depth *h* and thickness *t*:

2 $${\Psi }_{ij} = \mathop \sum \limits_{r = 1}^{M} \mathop \sum \limits_{s = 1}^{N} {\Psi }_{ij}^{rs} \left[ {v, \left( {h_{p} } \right)_{rs} , \left( {t_{p} } \right)_{rs} , a, b} \right]\;\;\;\;\;i, r = \left\{ {1, \ldots , M} \right\}; j, s = \left\{ {1, \ldots , N} \right\}.$$

Therefore, the inverse problem here consists of inverting ${\Psi }_{ij}$ to find the prism thickness ${\left({t}_{p}\right)}_{ij}$, since they are linearly related to the depth to the top of the prism. The estimation of ${\left({h}_{p}\right)}_{rs}$ and *v* can be achieved by deriving the analytical expression of the Fourier transform of ${\Psi }_{ij}$:

3 $${\stackrel{\sim }{\Psi }}^{rs}\left(\alpha ,\beta \right)=v {\stackrel{\sim }{\Phi }}^{rs}(\alpha ,\beta )$$

where *α* and *β* are the wavenumbers and ${\stackrel{\sim }{\Phi }}^{rs}$ is the Fourier transform of the apparent density function of unit-density.

Then, the inverse Fourier transform of ${\stackrel{\sim }{\Phi }}^{rs}$ at the center of each prism is expressed by:

4 $${\Phi }^{ij}\left({x}_{i},{y}_{j}\right)=\frac{2}{\pi }\sum_{k=1}^{\infty }\left[{tan}^{-1}\left(\frac{ab}{{t}_{k}\sqrt{{t}_{k}^{2}+{a}^{2}+{b}^{2}}}\right)-{tan}^{-1}\left(\frac{ab}{{\eta }_{k}\sqrt{{\eta }_{k}^{2}+{a}^{2}+{b}^{2}}}\right)\right]$$

where

5 $$\begin{gathered} t_{k} = kt - \left( {t_{p} } \right)_{rs} - w_{rs} + q_{rs} \hfill \\ \eta_{k} = kt - w_{rs} + q_{rs } \hfill \\ k = \left\{ {1, \ldots ,\infty } \right\} \hfill \\ \end{gathered}$$

and ${w}_{rs}$ is the depth to the bottom while ${q}_{rs}$ is the observation point altitude, which is both provided as a-priori information. Thus, the forward problem can be finally expressed as:

6 $${\Psi }_{ij} = v \mathop \sum \limits_{r = 1}^{M} \mathop \sum \limits_{s = 1}^{N} {\Phi }_{ij}^{rs} \left[ { \left( {t_{p} } \right)_{rs} } \right]\;\;\;\;\;i,r = \left\{ {1, \ldots ,M} \right\}; j,s = \left\{ {1, \ldots ,N} \right\}$$

A non-linear inverse approach can be adopted to estimate the thickness ${\left({t}_{p}\right)}_{rs}$ of each prism by transforming the above system to a set of *M* × *N* independent equations. Then, Eq. 6 can be written as:

7 $${\Psi }_{ij} = v\overline{\lambda }_{ij} {\overline{\Phi }}_{ij}^{rs} \left[ {\left( {t_{p} } \right)_{ij} } \right]\;\;\;\;\;i = \left\{ {1, \ldots ,M} \right\}; j = \left\{ {1, \ldots ,N} \right\}$$

where ${\stackrel{-}{\Phi }}_{ij}^{rs}$ is the ‘thickness estimator’ and ${\stackrel{-}{\lambda }}_{ij}$ is the ‘similarity function’ representing the degree of correlation between ${\Psi }_{ij}$ and ${\stackrel{-}{\Phi }}_{ij}^{rs}$. Therefore, the thickness estimator is computed from Eq. 4 for a set of ${\left({t}_{p}\right)}_{ij}$ and the solution that satisfies the a-priori constraints is that producing the highest correlation between ${\stackrel{-}{\Phi }}_{ij}^{rs}$ and ${\Psi }_{ij}$.

### Spectral methods of potential field data for depth estimation

Different spectral techniques have been proposed for depth estimation of potential field data, assuming either a statistical ensemble of blocks^62–64^, a random source distribution (e.g.^65–69^), or even a fractal source distribution (e.g.^70–75^). In particular, using the statistical mechanics postulate, it is showed that the mathematical expectation of an ensemble power density function is equal to the ensemble average^62^, and obtained an expression for computing the radial average power spectrum $\overline{E}(k)$:

8 $$\langle \overline{E}(k)\rangle =4{\pi }^{2}{\overline{M}}^{2}\langle {e}^{-2hk}\rangle \langle {T}^{2}(k)\rangle \langle {R}_{T}^{2}\rangle \langle {R}_{M}^{2}\rangle \langle {S}^{2}(k,a,b)\rangle$$

where *k* is the wavenumber, ${\overline{M}}^{2}$ is ﻿magnetic moment intensity, *T*^2^(*k*) is thickness factor, *h* is depth, *R*_*T*_ is a factor related to the geomagnetic field direction, *R*_*M*_ is a factor related to the magnetization direction, and ${S}^{2}(k,a,b)$ is a factor related to horizontal dimension sources (size factor). ${S}^{2}(k,a,b)$ was shown to follow a power-law form ${k}^{-\beta }$, with the decaying exponent of about − 2.9 for intermediate-to-large values of *a*^63^*.* With this correction, Eq. 8 becomes:

9 $$\mathit{ln}(\langle \overline{E}\left(k\right)\rangle )\approx \mathit{ln}(A)-2k\stackrel{-}{h}-\beta k$$

where *A* is a constant and $\beta =2.9$. Equation 9 is used to estimate average depth to the top of magnetic and gravity sources from the slope of the power spectrum at mid to high wavenumbers.

Regarding the depth to the source centroid^66,108^, we assume the spectrum from random and uncorrelated sources within a flat layer:

10 $$E(k)=B{e}^{-2k{h}_{o}}{\left({e}^{-k({h}_{t}-{h}_{o})}-{e}^{-k({h}_{b}-{h}_{o})}\right)}^{2}$$

where *B* is a constant, *h*_*t*_ is depth to the top and *h*_*o*_ the depth to the centroid. At long wavelengths, the estimate of the depth to the centroid is obtained from the slope of the azimuthally averaged wavenumber-scaled Fourier spectrum:

11 $$ln(E(k)/{k}^{2})=lnB-2k{h}_{o}$$

The depth to the bottom of the anomalous sources can then be easily computed as^66,78^:

12 $${h}_{b}=2{h}_{o}-{h}_{t}$$

In practice, spectral methods provide valid results if the optimal window size is chosen for the range of presumed depths^62,74^.

In this study we adopted different window sizes centered over different geological provinces. We choose windows with variable size, mainly because the geologic setting of Sicily varies significantly.

The standard error of the top and the centroid depths may be computed separately as^109^:

13 $$\varepsilon =\sqrt{\frac{1}{(n-2)}\left(\frac{{\sum }_{i=1}^{n}{\left({P}_{i}- \overset{\frown}{P} _{i}\right)}^{2}}{{\sum }_{i=1}^{n}{\left({k}_{i}-\overline{k}\right)}^{2}}\right)}$$

where $n$ is the number of observations, ${P}_{i}$ and $\overset{\frown}{P} _{i}$ are the observed and estimated values of the corrected power spectrum, ${k}_{i}$ and $\overline{k}$ are observed and mean values of the wavenumber considered for depth estimation.

Both depths to the Curie isothermal surface and Moho boundary depend on the estimated depth to the crystalline top as well as on the depth to its centroid.

The standard error of the depth to the Curie isothermal surface and Moho boundary may then be computed using:

14 $$\varepsilon =2{\varepsilon }_{o}+{\varepsilon }_{t}$$

where ${\varepsilon }_{o}$ and ${\varepsilon }_{t}$ are the standard errors of the crystalline centroid and top depths, respectively^109,110^.

## Supplementary information

Supplementary Information.

## Data Availability

The complete gravity and aeromagnetic datasets of Italy are available at the ISPRA website: https://portalesgi.isprambiente.it/en/elenco-base-dati/15.
