# Supplementary material for: Crustal structure of Sicily from modelling of gravity and magnetic anomalies
Source: Sci Rep. 2020 Sep 29;10:16019. doi: 10.1038/s41598-020-72849-z (PMC7525570; doi:10.1038/s41598-020-72849-z)
Supplement: Supplementary file 1 — Supplementary Information. [file 41598_2020_72849_MOESM1_ESM.pdf]

Crustal structure of Sicily from modelling of gravity and magnetic anomalies

M. Milano[1], Y. Kelemework[2*], M. La Manna[2], M. Fedi[2], D. Montanari[3], M. Iorio[1]

[1] Institute of Marine Sciences, National Research Council of Italy (CNR – ISMAR), Napoli, Italy.

[2] University of Naples Federico II, DiSTAR, Napoli, Italy.

[3] Institute of Geosciences and Earth Resources, National Research Council of Italy (CNR-IGG), Florence, Italy.

*Corresponding author: Yemane Kelemework (yemanekelemework.equbamariam@unina.it)

SUPPLEMENTARY INFORMATION

Figure S1. Examples of spectra for the estimation of the depth to the Crystalline top, Curie-isotherm, and Moho depth from offshore Sicily channel.

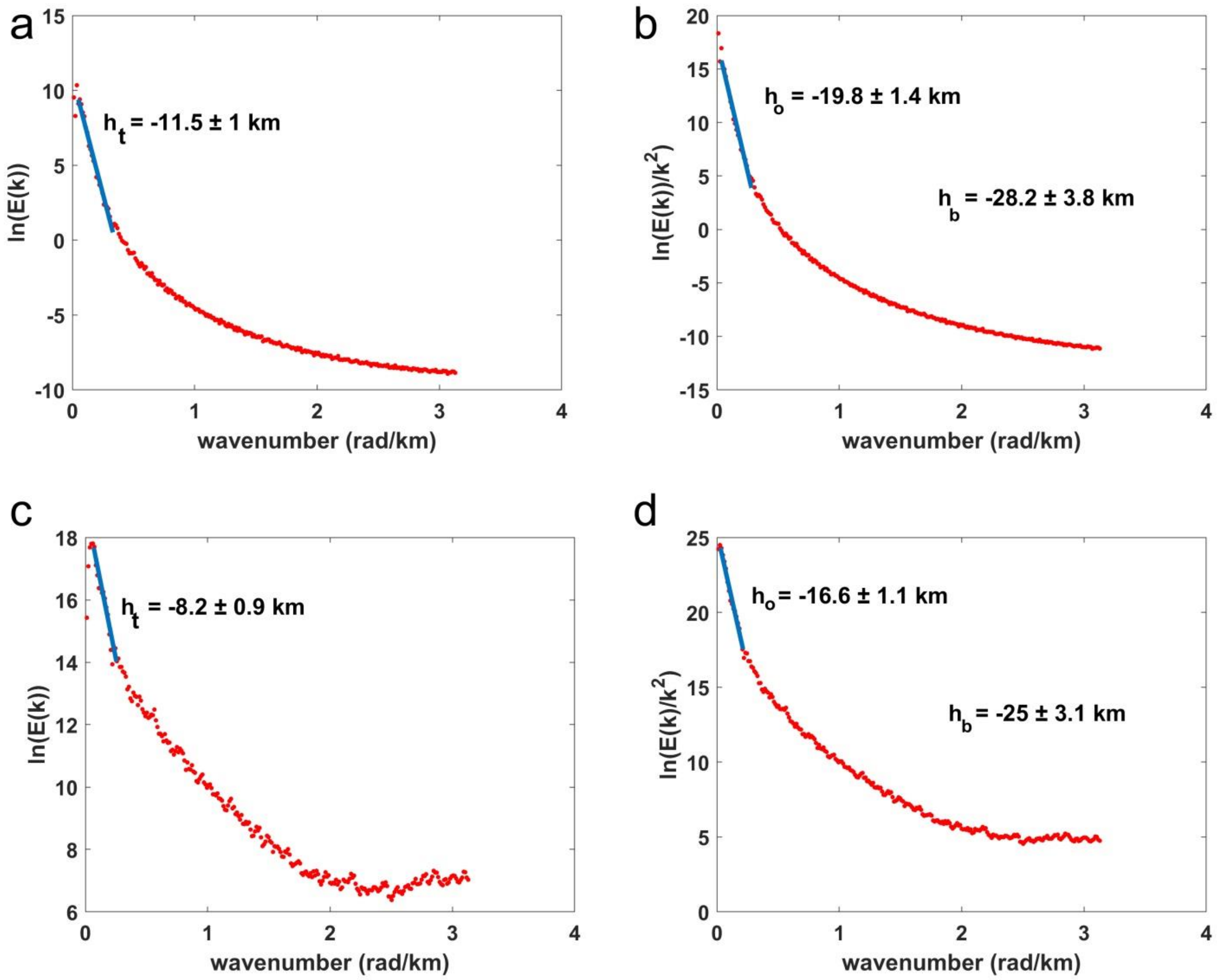

Figure S2. Comparisons of the estimated depth models with two seismic sections (a). The carbonate top, crystalline basement, Moho boundary and Curie isotherm surface are extrapolated along 2D profiles and plotted along the seismic interpretations of SI.RI.PRO [modified after [1]] (b) and CROP M-25 [modified after [2]] (c) seismic transects.

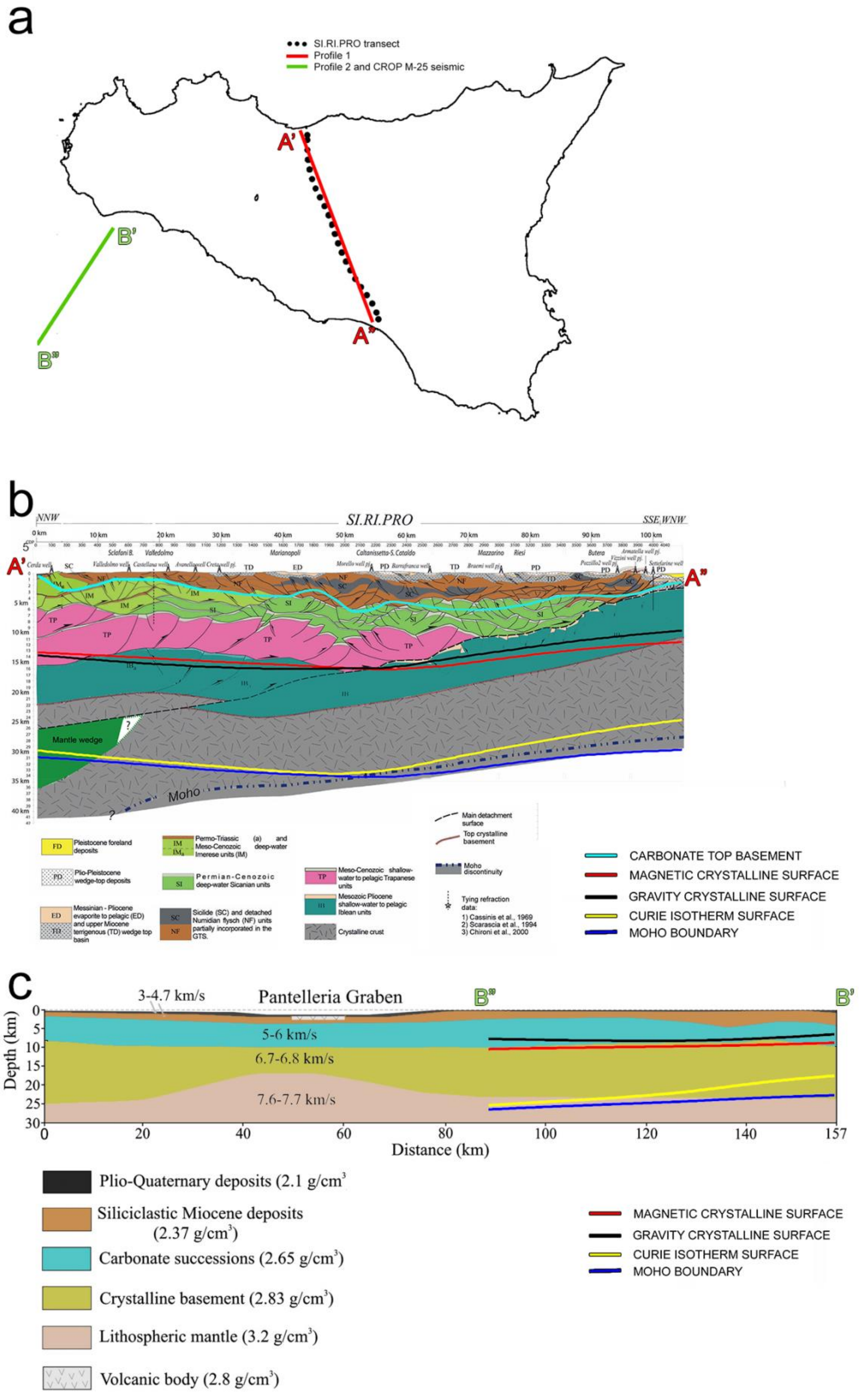

References

1. Catalano, R., Valenti, V., Albanese, C., Accaino, F., Sulli, A., Tinivella, U.,...Giustiniani, M. (2013b). Sicily's fold–thrust belt and slab roll-back: the SI.RI.PRO. seismic crustal transect. Journal of the Geological Society,170, 451–464.doi:10.1144/jgs2012-099.
2. Civile, D., Lodolo, E., Tortorici, L., Lanzafame, G., & Brancolini, G. (2008). Relationships between magmatism and tectonics in a continental rift: The Pantelleria Island region (Sicily Channel, Italy). Marine Geology, 251(1), 32–46.
